# Sequence Conservation and Sexually Dimorphic Expression of the *Ftz-F1* Gene in the Crustacean *Daphnia magna*

**DOI:** 10.1371/journal.pone.0154636

**Published:** 2016-05-03

**Authors:** Nur Syafiqah Mohamad Ishak, Yasuhiko Kato, Tomoaki Matsuura, Hajime Watanabe

**Affiliations:** 1 Department of Biotechnology, Graduate School of Engineering, Osaka University, Suita, Osaka, Japan; 2 Frontier Research Base of Global Young Researchers, Graduate School of Engineering, Osaka University, Suita, Japan; Leibniz Institute on aging—Fritz Lipmann Institute (FLI), GERMANY

## Abstract

Identifying the genes required for environmental sex determination is important for understanding the evolution of diverse sex determination mechanisms in animals. Orthologs of *Drosophila* orphan receptor Fushi tarazu factor-1 (Ftz-F1) are known to function in genetic sex determination. In contrast, their roles in environmental sex determination remain unknown. In this study, we have cloned and characterized the *Ftz-F1* ortholog in the branchiopod crustacean *Daphnia magna*, which produces males in response to environmental stimuli. Similar to that observed in *Drosophila*, *D*. *magna Ftz-F1* (*DapmaFtz-F1*) produces two splicing variants, *αFtz-F1* and *βFtz-F1*, which encode 699 and 777 amino acids, respectively. Both isoforms share a DNA-binding domain, a ligand-binding domain, and an AF-2 activation domain and differ only at the A/B domain. The phylogenetic position and genomic structure of *DapmaFtz-F1* suggested that this gene has diverged from an ancestral gene common to branchiopod crustacean and insect *Ftz-F1* genes. qRT-PCR showed that at the one cell and gastrulation stages, both *DapmaFtz-F1* isoforms are two-fold more abundant in males than in females. In addition, in later stages, their sexual dimorphic expressions were maintained in spite of reduced expression. Time-lapse imaging of *DapmaFtz-F1* RNAi embryos was performed in H2B-GFP expressing transgenic *Daphnia*, demonstrating that development of the RNAi embryos slowed down after the gastrulation stage and stopped at 30–48 h after ovulation. *DapmaFtz-F1* shows high homology to insect *Ftz-F1* orthologs based on its amino acid sequence and exon-intron organization. The sexually dimorphic expression of *DapmaFtz-F1* suggests that it plays a role in environmental sex determination of *D*. *magna*.

## Introduction

Sex determination is a fundamental biological process that governs the development of sexual characteristics, including the sexual differentiation of gonads, and affects the sexually dimorphic behavior, physiology, and morphology. The mechanism can be broadly categorized into two groups according to their primary causal factors: genetic sex determination (GSD) and environmental sex determination (ESD) [1–3]. In GSD, the sex-specific developmental pathway is resulted from the genetic segregation of genes, usually positioned on sex chromosomes. The ESD, however, relies on the environmental cues such as temperature, photoperiod, nutrition, and population density to induce molecular cascades for activation of alternate sex-determining genes [4, 5]. Currently, while GSD mechanism is well reported, the molecular basis of ESD has not yet been clarified. Analyzing the function of genes involved in ESD and unraveling the sex-determining pathways is crucial to understanding the origin and evolution of sex-determining pathways.

The water flea *Daphnia magna*, a crustacean living in freshwater ponds, undergoes switching of its reproductive strategy between asexual and sexual reproduction, depending on the environmental conditions [6]. Healthy *D*. *magna* produce female offspring by parthenogenesis or asexual cycle. Alternatively, in response to environmental stimuli such as insufficient food, short photoperiod and/or increased population density, it produces males, which allows for the fertilization of haploid eggs by sexual reproduction to produce resting eggs that can survive in harsh conditions [7,8]. The environmentally dependent production of males is a key process in the life cycle of *Daphnia* and leads to increased genetic diversity and fitness to overcome adverse conditions necessary for survival [9].

For male production in *D*. *magna* ESD, juvenile hormone (JH) and the DM domain gene *DapmaDsx1* are currently known to be essential. JH stimulates germ cells at the late stage of oogenesis leading to the development of males from ovulated eggs [10–13]. In response to the JH signal, *DapmaDsx1* is expressed and maintained to regulate development of male traits during embryogenesis [14], suggesting that JH-dependent *DapmaDsx1* activation is necessary for the environmentally dependent production of males. However, genes that mediate JH signaling and *DapmaDsx1* activation remain unknown.

Fushi tarazu factor-1 (Ftz-F1) is a member of the orphan nuclear receptor family involved in the genetic regulation of various developmental processes and was first identified in *Drosophila melanogaster* [15]. Subsequently, Ftz-F1 orthologs have been isolated from a wide range of animals and have several different names including steroidogenic factor-1 (Sf-1) [16], adrenal-4-binding protein (Ad4BP) [17] and nuclear hormone receptor-25 (nhr-25) [18]. The vertebrate *Ftz-F1* orthologs, *Sf-1* genes, are strongly linked to steroid biosynthesis and sex-determination pathways. In mammals, *Sf-1* genes are expressed in steroidogenic tissues, play a key role in regulating steroidogenesis, and are involved in the testis-determining pathway during genetic sex determination [19,20]. Recent studies found that *Drosophila* Ftz-F1 participates in JH signaling by interacting with Methoprene-tolerant (MET), a hormone receptor protein that directs JH-mediated gene activation [21,22]. Therefore, it is reasonable to hypothesize that the *Ftz-F1* ortholog may be a factor that mediates JH signaling and environmental sex determination in *Daphnia*.

In this study, we identified a *D*. *magna Ftz-F1* ortholog (*DapmaFtz-F1*) that produces two splicing variants, *αFtz-F1* and *βFtz-F1*, both of which exhibited sexual dimorphism in their expression during embryogenesis. Our findings suggest that *DapmaFtz-F1* is possibly required for male production in *D*. *magna* ESD.

## Materials and Methods

### *Daphnia* Strain and Culture Conditions

The *Daphnia magna* strain (NIES clone) was obtained from the National Institute for Environmental Studies (NIES; Tsukuba, Japan) and maintained as previously described [23] using ADaM [24] as the culture medium. Transgenic *D*. *magna* that exhibits ubiquitous and constitutive expression of GFP under the control of *D*. *magna* elongation factor 1 α-1 (EF1α-1) gene promoter [25] was used for microinjection experiments. To obtain male embryos, adult daphnids (2–3 weeks old) were treated with 1 μg/L of synthetic juvenile hormone analog, Fenoxycarb (Wako Pure Chemical; Osaka, Japan) [12]. Then, the ovulated eggs were collected and used for subsequent experiments.

### Cloning of the *D*. *magna Ftz-F1* Gene

Male and female daphnids were collected separately and briefly washed. Homogenization was performed with beads using a Micro Smash machine MS-100 (TOMY; Tokyo, Japan) in the presence of Sepasol-RNA I reagent (Nacalai Tesque Inc.; Kyoto, Japan). Total RNA was isolated according to the manufacturer’s protocol, which was followed by phenol/chloroform extractions. The purified total RNA was converted to first strand cDNA with SuperScript III Reverse Transcriptase (Invitrogen; Carlsbad, CA, USA), utilizing random primers (Invitrogen) according to the manufacturer’s recommended protocol. *DapmaFtz-F1* cDNA fragments that code for the DNA-binding domain (DBD) and the ligand-binding domain (LBD) were obtained from female cDNA by PCR with AmpliTaq DNA polymerase (Applied Biosystems; Foster City, CA, USA) using degenerate primers that were designed based on the conserved amino acid sequences of the DBD (5′-GAAGAACTGTGTCCNGTBTGYGG-3′) and the LBD (5′-ARTTTCATYTGRTCGTCAACCTT-3′). Amplified DNA fragments were cloned into a pGEM-T Easy Vector System (Promega Corp.; Madison, WI, USA) and sequenced.

Full-length cDNAs were completed by 5′ and 3′ rapid amplification of cDNA ends (RACE) with a GeneRacer Kit (Invitrogen) and a SMARTer RACE cDNA Amplification Kit (Clontech Laboratories Inc.; Mount View, WI, USA), respectively. The primer sequences for the RACE experiments were as follows: 5′-RACE gene specific primer (5′-TCCTCCGCCGGACGGGTGATTATTTG-3′); 5′-RACE nested primer (5′-TTCGCGGATCAATGGCGGAACTTTAGC-3′); 3′-RACE gene specific primer (5′-ATTCTCCGTCCGGCAGCAGCGTCTAC-3′); and 3′-RACE nested primer (5′-TCCACTTGCCGCATCACTCGGCTATC-3′). These amplification products were purified from an agarose gel after electrophoresis and were cloned into a TOPO vector, using a TOPO cloning kit (Invitrogen) for sequencing. The sequencing reaction was performed using a BigDye Terminator Cycle Sequencing Kit (PE Applied Biosystems, Foster City, CA, USA) and the DNA sequences were analyzed using the BLAST program.

### Phylogenetic Analysis of the *D*. *magna Ftz-F1* Gene

Amino acid sequences of *Ftz-F1* family genes were retrieved from the NCBI database (http://www.ncbi.nlm.nih.gov/) as shown Table 1, and the whole amino acid sequences of each protein were used to construct the phylogenetic tree. Multiple sequence alignments, based on the amino acid sequences, were constructed using the Clustal W [26] in MEGA version 6.06 [27]. The following settings were used for the analysis: pairwise alignment parameters: gap opening penalty = 6.00, gap extension penalty = 0.21, and identity protein weight; matrix multiple alignment parameters: gap opening penalty = 10.00, gap extension penalty = 0.24, delay divergent cut-off = 30%, and gap separation distance = 4. The phylogenetic reconstruction was performed using the p-distance algorithm and the neighbor-joining method implemented in MEGA.

**Table 1:** Accession numbers of Ftz-F1 ortholog genes used in this study.

Common name	Scientific name	Gene name (definition in NCBI)	Accession no.
Water flea	*Daphnia magna*	Ftz-F1	This work (LC105700, LC105701)
Water flea	*Daphnia pulex*	Ftz-F1	EFX77612.1
German cockroach	*Blattella germanica*	Ftz-F1	CAQ57670.1
Red flour beetle	*Tribolium castaneum*	Ftz-F1	EFA01263.1
Yellow fever mosquito	*Aedes aegypti*	Ftz-F1	AAF82307.1
Silkworm	*Bombyx mori*	Ftz-F1	NP_001037528.2
Shrimp	*Metapenaeus ensis*	Ftz-F1	AAD41899.1
Fruit fly	*Drosophila melanogaster*	Ftz-F1	AAA28542.1
Mouse	*Mus musculus*	Sf-1	AAB28338.1
Zebrafish	*Danio rerio*	Nr5a2	NP_571538.1
Medaka	*Oryzias latipes*	Ftz-F1	BAA32394.1
Roundworm	*Caenorhabditis elegans*	Nhr-25	CAA91028.1

### Temporal Expression Analysis by Quantitative Real-Time PCR

Male and female embryos were collected at 0, 6, 12, 18, 24, 30, 48, and 72 h after oviposition. These time points correspond to several embryonic stages described in [25,28,29]. To have three biological replicates, the collected embryos at each stage were divided into three groups. Each group was subjected to total RNA purification as described above. The number of embryos and amounts of purified total RNAs in each group were shown in S1 Table. cDNAs were synthesized using 1 μg of total RNAs as mentioned above. Of each cDNA pool, 1/120 volume was used as a template for qRT-PCR, providing us an equation (1) for calculation of number of embryos subjected to qRT-PCR.

N={1(μg)/[(amounts(μg)of purified RNAs)/(number of embryos)]}/120 (1)

**Fig 1:**
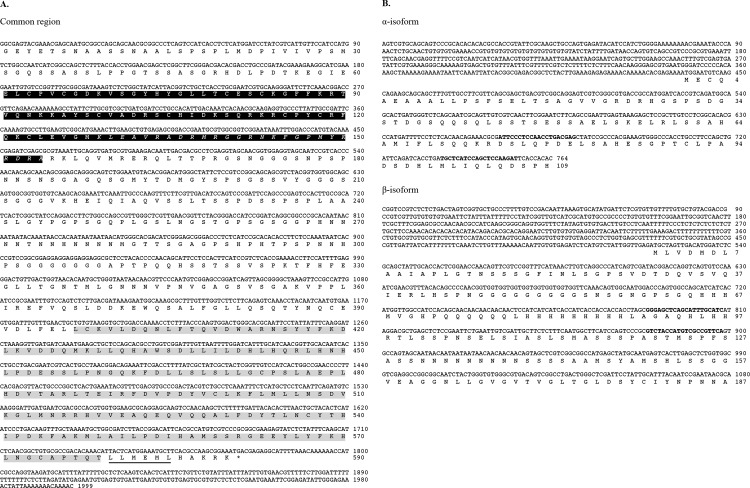
Nucleotide and deduced amino acid sequences of *D*. *magna Ftz-F1*. (A) The *DapmaFtz-F1* common region. Black and grey shaded amino acids indicate the putative DNA-binding domain (DBD) and ligand-binding domain (LBD) respectively, based on the alignment of amino acid sequences of signature domains. The Ftz-F1 box (italicized) and the activation factor-2 (AF-2) core (underlined) motifs are indicated. (B) Nucleotide sequence of *DapmaFtz-F1* isoform-specific regions. Deduced amino acid sequences starting from the first methionine for each isoform are also indicated. Locations of the primers used in qRT-PCR are emboldened. Numbers on the right indicate the nucleotide and amino acid positions.

PCR was performed using a SYBR GreenER qPCR SuperMix Universal Kit (Invitrogen) with Mx3005P Real-Time PCR System (Agilent Technologies; CA, USA). In the presence of the appropriate primer pairs, real-time PCR amplifications were performed in triplicate at the following conditions: 2 min at 50°C and 10 min 95°C, followed by 40 cycles of 15 s at 95°C and 1 min at 60°C. Gel electrophoresis and dissociation curve analyses were performed to confirm the correct amplicon size and the absence of non-specific bands. Copy number of *DapmaFtz-F1* mRNAs was measured by the quantification method, which relates the PCR signal to the input copy number by using a calibration curve obtained by a dilution series of plasmid that contains sequences corresponding to each primer set. Finally, copy number obtained by qRT-PCR was divided by *N*, resulting in copy number of transcripts in one embryo. The oligonucleotide sequences for qRT-PCR are shown in Fig 1B.

### Gene Function Analysis by RNA Interference

RNA-mediated interference with 100 μM of Ftz-F1_699 siRNA (5′-CCAGUCUCUGACGAUAA-3′) and Ftz-F1 918 siRNA (5′-GCACACACCUUCUCCAAAU-3′) were employed to knock down *DapmaFtz-F1* function *in vivo* by the method of microinjection into *Daphnia* eggs [30]. Eggs were obtained from a *D*. *magna* transgenic line at 2–3 weeks of ages, directly after the ovulation and placed in ice-cold M4 media that contained 80 mM sucrose. The injection solutions contained the specified siRNAs, mixed with 0.02 μM Alexa Fluor 568 dye (LifeTechnologies Inc.; Grand Island, NY, USA) as a marker to check whether an appropriate volume of solution was injected. The injected eggs were incubated in a 96-well plate at 23°C. A random sequence (5′-GGUUAAGCCGCCUCACAUTT-3′) that did not affect *Daphnia* embryogenesis [31] was utilized as a control siRNA (Control_416 siRNA). The phenotypes of injected embryos were carefully observed by time-lapse imaging from 3 h to 30 h after ovulation by fluorescence microscopy. At 24 h after injection, total RNAs were isolated from two embryos injected with Control_416 or Ftz-F1_918 siRNA in three replicates and were converted to cDNAs as described above. RT-qPCR analysis was performed with the same protocol mentioned above except that two primers, the FTZ-F1-realtime-5 (5′-CGCACACCTTCTCCAAATAA-3′) and FTZ-F1-realtime-3 (5′-TTACCAGTCAACAGTCCCTCAAAA-3′) were used to amplify the common region of *Ftz-F1* gene.

## Results and Discussion

### Characterization of cDNAs Encoding *D*. *magna Ftz-F1*

To examine the existence of the *Ftz-F1* ortholog in *D*. *magna*, we designed degenerate primers for amplification of the *DapmaFtz-F1* cDNA fragment that codes for the DBD and the LBD regions (Fig 1A). After cloning and sequencing the amplified DNA fragments, a BLAST analysis revealed that the putative amino acid sequence shows high homology to *Dr*. *melanogaster* nuclear hormone receptor Ftz-F1. Therefore, we designated this gene as *DapmaFtz-F1* (i.e., *D*. *magna Ftz-F1* gene).

To identify full-length *DapmaFtz-F1* cDNA, 5′ and 3′ RACE reactions were performed using cDNAs of male and female adults. The sequences were assembled into two different isoforms, *αFtz-F1* and *βFtz-F1*, which are composed of 2,763 and 3,078 nucleotides, respectively. The open reading frames (ORFs) for *αFtz-F1* and *βFtz-F1* encode 699 and 777 amino acid residues respectively. They differed at the 5′ UTR and 5′ region of the ORF (Fig 1B). No sex-specific transcript was found from the RACE experiments.

### Features of *D*. *magna* Ftz-F1 Proteins

**Fig 2:**
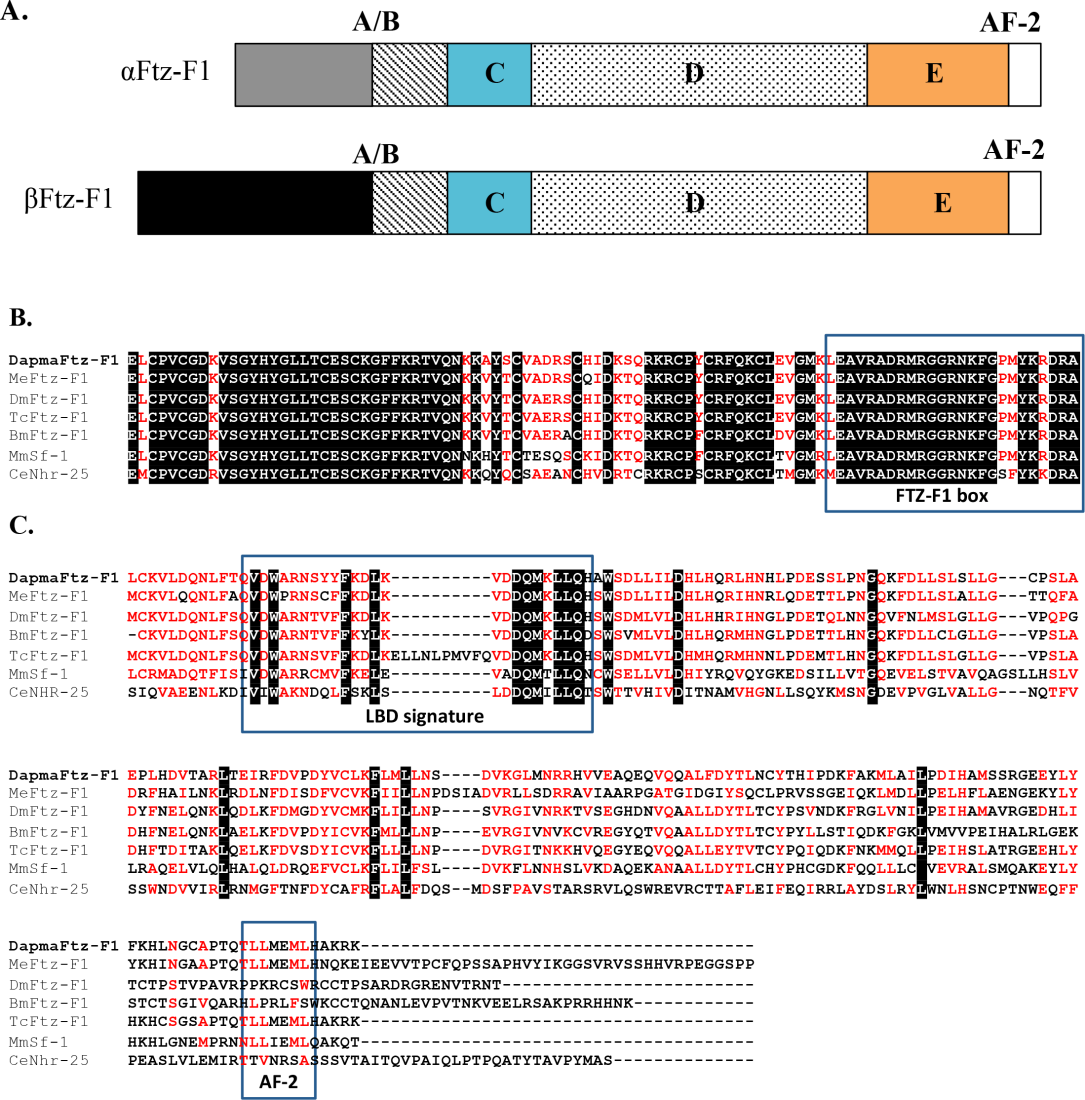
Evolutionary conserved domains of *D*. *magna* Ftz-F1. (A) Schematic diagram of the Ftz-F1 regions that are divided into A/B, C, D and E regions. (B) Alignment of the C region and the Ftz-F1 box (boxed), and (C) alignment of the E region showing the LBD signature domain (boxed) and AF-2 motif (boxed). Identical amino acids are shaded in black whereas amino acids with similar characteristics are colored in red. MeFtz-F1 is the *Me*. *ensis* (shrimp) protein; DmFtz-F1 is the *Dr*. *melanogaster* (fruit fly) protein; BmFtz-F1 is the *B*. *mori* (silkworm) protein; and TcFtz-F1 is the *T*. *castaneum* (beetle) protein. MmSf-1 is the steroidogenic factor-1 of *Mu*. *musculus* (mouse); and CeNhr-25 is the nuclear hormone receptor-25 of *C*. *elegans* (roundworm).

We compared amino acid sequences of DapmaFtz-F1 proteins with those of Ftz-F1 orthologs from *Drosophila* and various animals. The multiple alignment revealed that DapmaFtz-F1 proteins were predicted to have the typical structure of a nuclear receptor, which consists of an A/B region, a conserved zinc finger DBD at a DNA sequence recognition C region, a hinge D region, and lastly, an LBD follow by an activation function-2 (AF-2) at the E region (Fig 2A). Both αFtz-F1 and βFtz-F1 proteins consist of identical 590 amino acid sequences, which include the DBD (94 aa) and the LBD (182 aa) regions (Fig 1A). They have a different amino acid sequence at the A/B region, where αFtz-F1 and βFtz-F1 have 109 aa and 187 aa, respectively (Fig 1B). No conserved motif among Ftz-F1 orthologs was found in the A/B region of the α- or β-isoform. The DBD region and the LBD regions of DapmaFtz-F1 were aligned with other Ftz-F1 orthologs, namely *Metapenaeus ensis* Ftz-F1, *Dr*. *melanogaster* Ftz-F1, *Bombyx mori* Ftz-F1, *Tribolium castaneum* Ftz-F1, *Mus musculus* Sf-1, and *Caenorhabditis elegans* nhr-25 (Fig 2B and 2C). In the C region, a sequence named the Ftz-F1 box, adjacent to the zinc-finger motif [32], was conserved (Figs 1A and 2B). This DNA sequence recognition region is the most conserved region of the amino acid sequence. In the E region, the LBD signature and AF-2 motif that is required for ligand binding [32–34] was also found (Figs 1A and 2C). Both regions of *DapmaFtz-F1* were the most homologous to those of insect orthologs.

### Phylogenetic Analysis of *DapmaFtz-F1*

**Fig 3:**
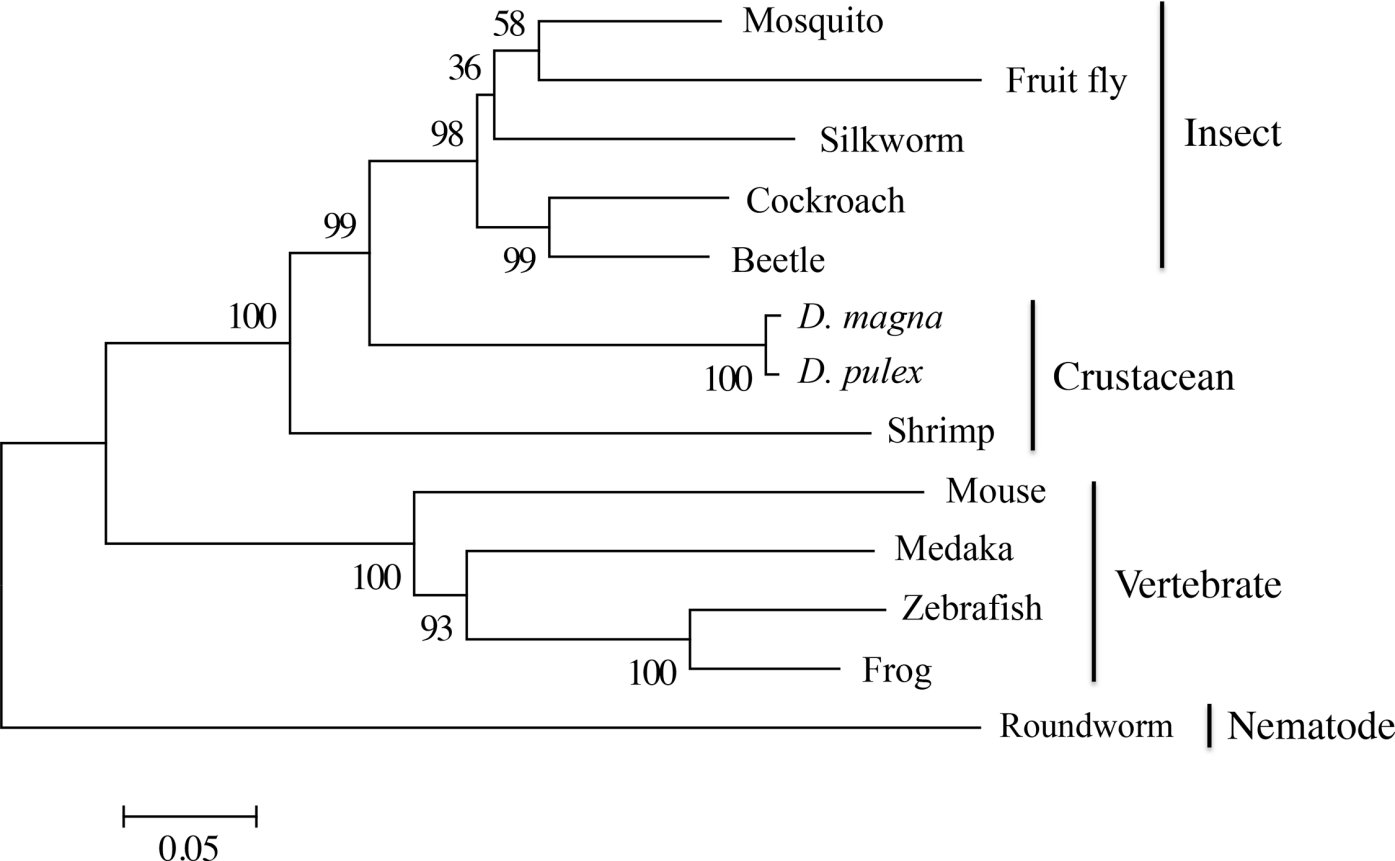
Phylogenetic tree of the amino acid sequences of the DBD and LBD Ftz-F1 nuclear hormone receptor subfamily. The percentages of the replicate tree in which the associated taxa clustered together in the bootstrap test (1,000 replicates) are shown next to the branches. The bar indicates branch length and corresponds to the mean number of the differences (*P<0*.*05*) per residue along each branch. Evolutionary distances were computed using the p-distance method.

To analyze the evolutionary relationship of *DapmaFtz-F1* further, a phylogenetic tree of *DapmaFtz-F1* with 12 other *Ftz-F1* related genes was constructed by the neighbor-joining method, using whole amino acid sequences (Fig 3). The topology of the phylogenetic relationship between Ftz-F1 orthologs was in good agreement with the taxonomic relationship between insects and crustaceans. Compared to the Ftz-F1 ortholog of a shrimp belonging to malacostracan crustaceans, the branchiopod crustacean *Daphnia* Ftz-F1 was more closely related to insect Ftz-F1 orthologs. Our result supports the hypothesis that insects originated from branchiopod crustaceans [35].

### Genomic Organization of the *DapmaFtz-F1* Gene

**Fig 4:**
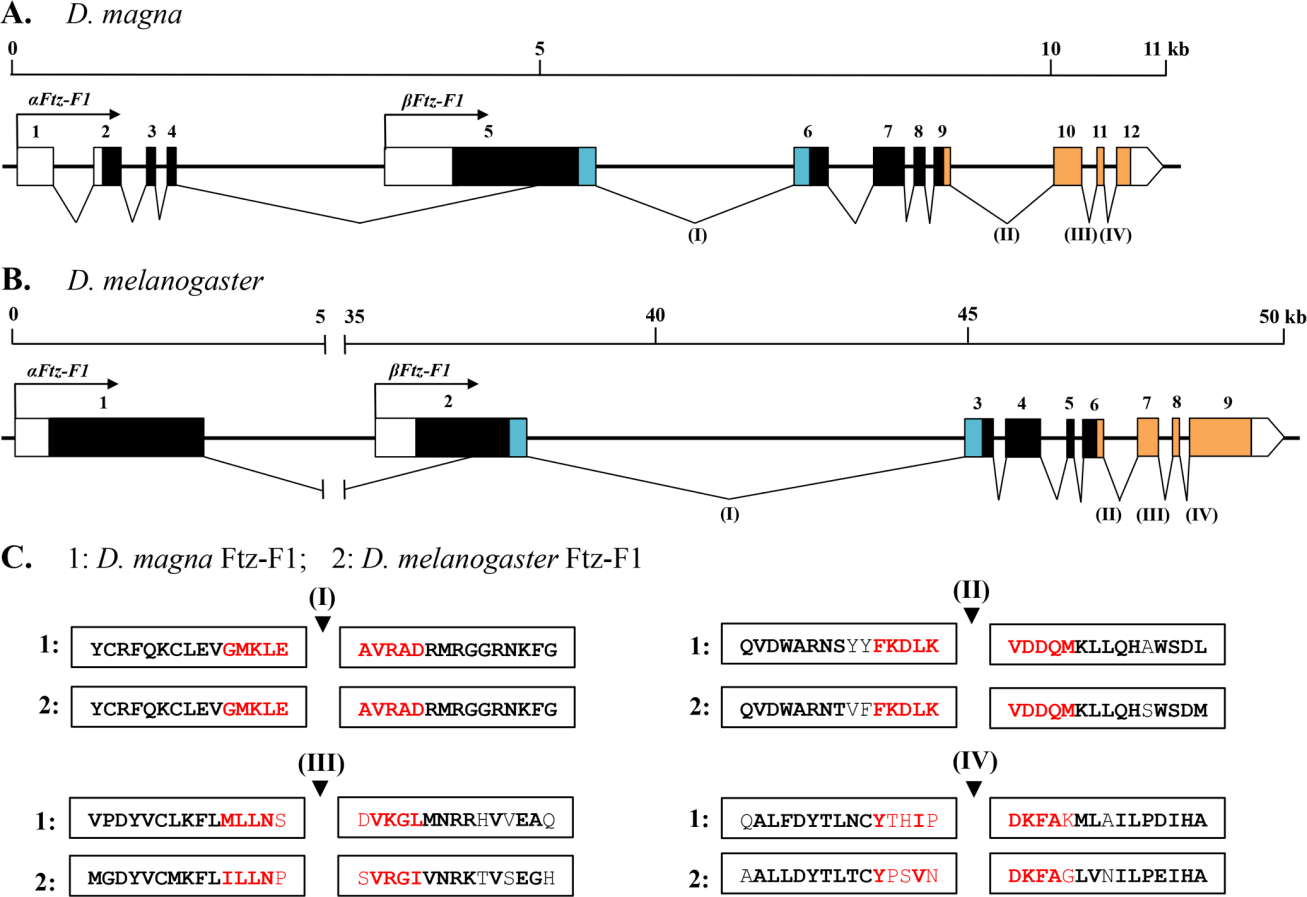
**Genomic structural organization of (A) the *D*. *magna Ftz-F1* gene and (B) the *Dr*. *melanogaster Ftz-F1* gene.** The numbered boxes are exons, and the intervening lines are introns. Colored boxes indicate coding regions; blue represents DBD, whereas orange represents LBD. Empty boxes indicate untranslated regions. Each isoform possesses a unique coding sequence at the 5′end, with black arrows indicating the transcription start site. Scale bars are provided at the top of each diagram for the size in kilobases. Numbers I, II, III, and IV indicate the location of intron splice sites that are conserved between *D*. *magna* and *Dr*. *melanogaster*. (C) Putative conserved splice sites mapped to the conserved domain of Ftz-F1 from (1) *D*. *magna* and (2) *Dr*. *melanogaster*. The amino acid sequences shown are from DBD (Number I) and LBD (Numbers II, III, and IV). Red amino acids indicate 10 residues (five residues for pre- and post-introns, respectively) around the intron position assigned as the splice site, whereas further homology up and downstream of the intron is represented in black. Bold amino acid residues are residues shared between two species. Black triangles indicate the location of the intron within the splice site.

Next, we mapped the *DapmaFtz-F1* transcripts to the genomic sequences and examined the exon-intron structure. The genomic structure of the *DapmaFtz-F1* gene is composed of 12 exons, spread over ~11 kb of genomic DNA as illustrated in Fig 4A. All exon-intron junctions possessed the consensus “GT-AG” nucleotides at their 5′ and 3′ splicing sites. *αFtz-F1* contains all 12 exons except a partial deletion of exon 5, whereas the *βFtz-F1* lacks exons 1–4. The region encoding the DBD is within two exons (i.e., exon 5 and 6) that are separated by a large intron of 2,006 bp, and the LBD region is located in four exons, which are exons 9–12.

The exon arrangement is similar to the structural organization of *Dr*. *melanogaster Ftz-F1* (Fig 4B) [36,37]. The common region between *αFtz-F1* and *βFtz-F1* is composed of eight exons and seven introns in both *D*. *magna* and *Dr*. *melanogaster*. Importantly, four of the seven intron positions are conserved between the two species (I, II, III, and IV in Fig 4C). The first position (I) is at the end of the C region just upstream of the Ftz-F1 box within the DBD. The other three intron positions (II, III, and IV) are located at the E region or LBD. Taken together with the result of the phylogenetic analysis, we speculate that *DapmaFtz-F1* has diverged from an ancestral gene common to branchiopod crustacean and insect *Ftz-F1* genes.

### *DapmaFtz-F1* mRNA Expression during Embryogenesis

**Fig 5:**
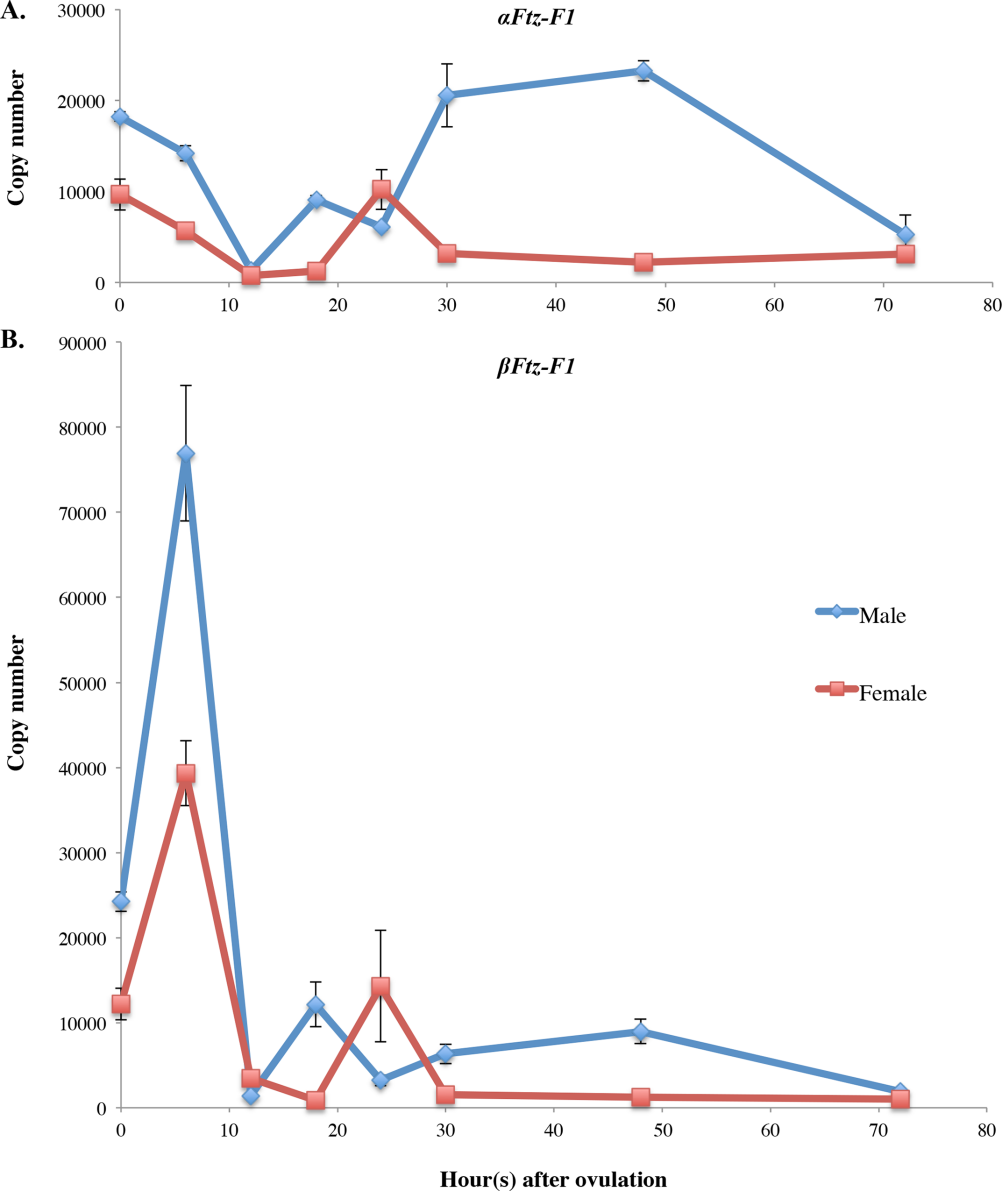
Temporal expression profiles of the *DapmaFtz-F1* gene in embryonic developmental stages of *D*. *magna*. (A) *DapmaFtz-f1 g*ene expression levels of the *α*-isoform and (B) the *β*-isoform in one embryo of males (blue line) and females (red line). Embryonic development was staged at 0 h (single cell egg), 6 h (late gastrulation stage), 12 h (cephalic-appendage developing stage), 18 h (early thoracic appendage-developing stage), 24 h (after hatching embryo), 30 h (middle carapace-developing stage), 48 h (further developed thoracic appendages and antennae embryo), and 72 h (juvenile *Daphnia*) after oviposition. Results are shown as copy numbers of transcript per egg. The copy numbers were measured from three independent qPCR amplifications and error bars represent standard error values across samples.

Because the genes related to sex determination and differentiation are known to show sex-specific differences in the abundance of transcripts [14], we next investigated the sexual differences of *DapmaFtz-F1* expression at various embryonic stages using a quantitative real-time PCR assay. Isoform-specific amplification was achieved by designing primers at the 5′ end of each coding region of *αFtz-F1* and *βFtz-F1* transcripts, as shown in Fig 1B. Adults were exposed to the JH agonist Fenoxycarb 9 h before oviposition, a critical stage for sex determination [13], inducing the ovulated eggs to develop as males. The eggs that developed as females were collected from unexposed mothers. The result of qRT-PCR analyses is presented in S1 File and Fig 5.

Just after oviposition (0 h), *αFtz-F1* expression in males was almost two-fold higher than in females. Between 0 and 12 h, *αFt-zF1* expression gradually decreased in both males and females. In the later embryonic stages (post 12 h) *αFtz-F1* was absent in females, except for a detectable peak at 24 h; in males it was expressed at each time point but was depleted by 72 h (Fig 5A), when the embryos become juveniles and swim out from their mother’s brood chamber. The temporal change in *βFtz-F1* expression was more prominent, especially during early embryogenesis (Fig 5B). At 0 h, the quantity of *βFtz-F1* mRNA was also two-fold higher in males. This isoform was activated three-fold at 6 h during the gastrulation stage and then dropped at 12 h in both males and females. During middle and late embryogenesis, the *β* isoform exhibited sexually dimorphic expression; however, its expression level was lower than that of the *α* isoform.

As summarized in Table 2, the *DapmaFtz-F1* transcripts are dominantly expressed in males at most embryonic stages, except at 24 h for the *αFtz-F1* isoform, and at 12 h and 24 h for the *βFtz-F1* isoform, suggesting that *DapmaFtz-F1* may play a role in regulating male trait development during embryogenesis.

**Table 2:** Sexual differences in *αFtz-F1* and *βFtz-F1* expression in *D*. *magna* during embryogenesis. *Ftz-F1* expression was normalized using *Ribosomal L32* expression as a reference gene. The fold difference was obtained by normalizing male expression to female expression.

Time after ovulation (h)	*αFtz-F1* expression		*αFtz-F1* expression	
	Fold difference	Std. dev.	Fold difference	Std. dev.
**0**	1.88	± 0.09	1.98	± 0.17
**6**	2.52	± 0.25	1.96	± 0.35
**12**	1.74	± 0.31	0.41	± 0.05
**18**	7.32	± 0.72	14.49	± 5.40
**24**	0.60	± 0.06	0.22	± 0.07
**30**	6.49	± 1.90	4.12	± 1.30
**48**	10.33	± 0.87	7.06	± 1.95
**72**	1.68	± 1.21	1.91	± 0.34

Std. dev. = standard deviation.

### Time-Lapse Imaging of *DapmaFtz-F1* RNAi Embryos

To examine the roles of the *DapmaFtz-F1* gene, *DapmaFtz-F1* expression was knocked down using the RNAi method [30] in male and female embryos. We used transgenic *Daphnia*, carrying the GFP gene fused to histone H2B gene [25], because the nuclear stain with the H2B-GFP protein enhances the visualization of cell dynamics in live embryos. Embryonic development was recorded by time-lapse imaging from 3 h to 30 h after ovulation. The development of knockdown embryos was similar to the control egg (non-injected) until the gastrulation stage (6 h). Subsequently, GFP intensity started to weaken and the development of RNAi embryos slowed down and did not develop normally (S1 Movie). All of the *Ftz-F1* siRNA-injected embryos failed to hatch, but their development slowly continued up to the eye developmental stage, because we observed that some of the injected embryos developed eye pigment after a 48 h incubation (Table 3). There was no difference in the RNAi phenotype between male and female embryos that were observed in this experiment.

**Table 3:** Summary of the siRNA microinjection experiment for phenotype observation. Excluding the Control_416 siRNA-injected embryos, all *Ftz-F1* siRNA-injected embryos developed abnormally, including failure to shed the outer egg membrane and slow development compared to that observed with normal eggs.

siRNA (100 μM)	Sex of egg	Number of injected eggs	Survived for 24 h	Developed eye pigment by 48 h
Control_416	Female	9	7	7
Control_416	Male	12	10 (*6)	4
Ftz-F1_699	Female	11	7	4
Ftz-F1_918	Female	10	8	8
Ftz-F1_918	Male	19	12 (*6)	6

* The embryos were subjected to total RNA isolation for qRT-PCR analysis.

To confirm that the *DapmaFtz-F1* expression has been successfully knocked down during RNAi, total RNA from 24 h siRNA injected-male embryos was isolated, and the *DapmaFtz-F1* expression level was measured. The qRT-PCR analysis (S2 File) showed that 90 ± 5% of expression was suppressed when compared to the control, indicating that RNAi effectively occurred in the siRNA-injected embryos. In short, the results suggested that *DapmaFtz-F1* was essential for embryonic development of *D*. *magna*.

Although embryonic lethality prevented us from analyzing sex-specific functions of *DapmaFtz-F1*, its expression pattern suggested three potential roles in *Daphnia* ESD. First, in one cell embryos at 0 h after ovulation, male embryos exhibited two-fold higher expression of both isoforms compared to that observed in females (Table 2), suggesting that JH-dependent activation of *DapmaFtz-F1* occurred during oogenesis, and the synthesized transcripts were deposited into eggs as maternal RNAs. This may imply that *DapmaFtz-F1* may be a direct target of JH, and these maternal transcripts may function in activating *DapmaDsx1* during male development. Consistent with this assumption, we found several candidates of Ftz-F1- binding site in the *DapmaDsx1* promoter (S3 File). Second, during the gastrulation stage, *βFtz-F1* was transiently activated and highly expressed in males (Fig 5). Importantly, this isoform was more abundant compared to the *αFtz-F1* transcript, suggesting that *βFtz-F1* may have a dominant role in sex determination at this stage. Third, in the later stages of embryogenesis, the expression of the *αFtz-F1* transcript was observed to be dominant, which may suggest that *αFtz-F1* is responsible for male trait development. In previous studies, the *Me*. *ensis* Ftz-F1 was also detected in the testis [34] and Ftz-F1α of *Xenopus laevis* was discovered in the developing gonads and testis [38]. In addition, the homolog of Ftz-F1 in mammalians, Sf-1, is a critical regulator of normal development of the hypothalamic-pituitary-gonadal axis during reproduction and sexual differentiation [20]. These findings indicated that the role of *Ftz-F1* orthologs in sexual development is evolutionarily conserved among species. To understand these possible functions of *DapmaFtz-F1*, more sophisticated methods for analyzing gene functions, such as tissue-specific and inducible knockdown methods, would be required in the future.

## Conclusion

In this study, we identified the *Ftz-F1* gene from *D*. *magna* and showed that *DapmaFtz-F1* is very closely related to insect *Ftz-F1* orthologs. Additionally, our study revealed that *DapmaFtz-F1* expression during embryogenesis is sexually dimorphic for both isoform variants. We speculate that DapmaFtz-F1 may have sex-specific functions in environmental sex determination of *D*. *magna*.

## Supporting Information

S1 File The raw data of qRT-PCR analyses for *αFtz-F1* and *βFtz-F1* expression. (DOCX)

S2 File The raw data of qRT-PCR analyses and normalized expression of *DapmaFtz-F1* in RNAi male embryos. (DOCX)

S3 File The candidates of Ftz-F1-binding site in the *Dsx1* promoter of *D*. *magna* and *D*. *pulex*. (DOCX)

S1 Movie Time-lapse imaging of *DapmaFtz-F1* RNAi embryos in G2B-GFP transgenic *Daphnia*. The development of three siRNA-injected embryos with one non-injected embryo as control (top) was recorded from 3–30 h after ovulation.
(AVI)

S1 Table The number of embryos and amount of isolated total RNA from each males and females sample. (DOCX)
