# Supplementary material for: Sequence Conservation and Sexually Dimorphic Expression of the Ftz-F1 Gene in the Crustacean Daphnia magna
Source: PLoS One. 2016 May 3;11(5):e0154636. doi: 10.1371/journal.pone.0154636 (PMC4854414; doi:10.1371/journal.pone.0154636)
Supplement: S1 File — (DOCX) [file pone.0154636.s001.docx]

**S1 - Supplementary Information**

**The raw data of qRT-PCR analyses for *αFtz-F1* and *βFtz-F1*** **expression.**

**Table 1:** The raw data *αFtz-F1* gene expression of each sample from qRT-PCR analysis.

| **Sex** | **Time (h)** | **Group 1** | **Group 2** | **Group 3** | **Average** | **Std. dev.** | **Std. e.** |
| --- | --- | --- | --- | --- | --- | --- | --- |
| **Male** | **0** | 4358.0 | 5024.0 | 5077.0 | 4819.7 | 400.7 | 231.3 |
|  | **6** | 4177.0 | 3344.0 | 4221.0 | 3914.0 | 494.1 | 285.3 |
|  | **12** | 394.7 | 317.9 | 533.6 | 415.4 | 109.3 | 63.1 |
|  | **18** | 1453.0 | 1519.0 | 1293.0 | 1421.7 | 116.2 | 67.1 |
|  | **24** | 703.5 | 740.1 | 901.5 | 781.7 | 105.4 | 60.8 |
|  | **30** | 1838.0 | 3644.0 | 3626.0 | 3036.0 | 1037.5 | 599.0 |
|  | **48** | 1902.0 | 1716.0 | 1660.0 | 1759.3 | 126.7 | 73.1 |
|  | **72** | 106.5 | 184.9 | 422.5 | 238.0 | 164.5 | 95.0 |
| **Female** | **0** | 3475.0 | 3629.0 | 2154.0 | 3086.0 | 810.8 | 468.1 |
|  | **6** | 2069.0 | 1221.0 | 1703.0 | 1664.3 | 425.3 | 245.6 |
|  | **12** | 283.7 | 241.8 | 184.7 | 236.7 | 49.7 | 28.7 |
|  | **18** | 341.9 | 242.1 | 302.5 | 295.5 | 50.3 | 29.0 |
|  | **24** | 2255.0 | 1060.0 | 1432.0 | 1582.3 | 611.5 | 353.1 |
|  | **30** | 436.2 | 382.0 | 337.8 | 385.3 | 49.3 | 28.5 |
|  | **48** | 261.4 | 215.6 | 169.5 | 215.5 | 46.0 | 26.5 |
|  | **72** | 190.8 | 85.2 | 82.5 | 119.5 | 61.8 | 35.7 |

**Table 2:** The raw data *βFtz-F1* gene expression of each sample from qRT-PCR analysis.

| **Sex** | **Time (h)** | **Group 1** | **Group 2** | **Group 3** | **Average** | **Std. dev.** | **Std. e.** |
| --- | --- | --- | --- | --- | --- | --- | --- |
| **Male** | **0** | 5534.0 | 7294.0 | 6470.0 | 6432.7 | 880.6 | 5534.0 |
|  | **6** | 18610.0 | 23620.0 | 20830.0 | 21020.0 | 2510.4 | 18610.0 |
|  | **12** | 382.0 | 395.3 | 570.4 | 449.2 | 105.1 | 382.0 |
|  | **18** | 1275.0 | 2896.0 | 1645.0 | 1938.7 | 849.5 | 1275.0 |
|  | **24** | 485.8 | 340.3 | 380.7 | 402.3 | 75.1 | 485.8 |
|  | **30** | 787.2 | 765.3 | 1177.0 | 909.8 | 231.6 | 787.2 |
|  | **48** | 847.7 | 509.7 | 668.9 | 675.4 | 169.1 | 847.7 |
|  | **72** | 80.7 | 80.6 | 101.7 | 87.7 | 12.2 | 80.7 |
| **Female** | **0** | 4694.0 | 4196.0 | 2860.0 | 3916.7 | 948.4 | 4694.0 |
|  | **6** | 12770.0 | 10720.0 | 11020.0 | 11503.3 | 1107.2 | 12770.0 |
|  | **12** | 995.0 | 1504.0 | 803.6 | 1100.9 | 362.0 | 995.0 |
|  | **18** | 183.6 | 173.2 | 244.8 | 200.5 | 38.7 | 183.6 |
|  | **24** | 4298.0 | 1257.0 | 1111.0 | 2222.0 | 1799.4 | 4298.0 |
|  | **30** | 286.5 | 103.4 | 161.0 | 183.6 | 93.6 | 286.5 |
|  | **48** | 116.5 | 128.3 | 122.3 | 122.4 | 5.9 | 116.5 |
|  | **72** | 44.7 | 33.0 | 37.0 | 38.2 | 5.9 | 44.7 |
