# Supplementary material for: Sequence Conservation and Sexually Dimorphic Expression of the Ftz-F1 Gene in the Crustacean Daphnia magna
Source: PLoS One. 2016 May 3;11(5):e0154636. doi: 10.1371/journal.pone.0154636 (PMC4854414; doi:10.1371/journal.pone.0154636)
Supplement: S2 File — (DOCX) [file pone.0154636.s002.docx]

**S2 - Supplementary Information**

**Table 1: The raw data from q-PCR analysis.** The *Ftz-F1* expression was normalized with reference gene *Ribosomal protein* *L32* [1].

| **SiRNA** | **Group** | **L32** | **Ftz-F1** | **Ftz-F1/ L32** | **Mean** | **Std. dev.** | **Std. e.** |
| --- | --- | --- | --- | --- | --- | --- | --- |
| **Control_416** | **1** | 1.35E+05 | 9.46E+03 | 7.03E-02 | 1.32E-01 | 0.057 | 0.033 |
|  | **2** | 1.30E+05 | 1.85E+04 | 1.42E-01 |  |  |  |
|  | **3** | 1.16E+05 | 2.14E+04 | 1.84E-01 |  |  |  |
| **Ftz-F1_918** | **1** | 3.80E+04 | 9.53E+02 | 2.51E-02 | 1.22E-02 | 0.012 | 0.007 |
|  | **2** | 1.21E+05 | 1.05E+03 | 8.70E-03 |  |  |  |
|  | **3** | 1.19E+05 | 3.37E+02 | 2.83E-03 |  |  |  |

**Figure 1: The normalized *DapmaFtz-F1* expression level of siRNA injected-male embryos 24 h after injection.** The *Ftz-F1* expression was normalized using *Ribosomal protein L32* expression as a reference gene and the expression from Control_416 was set as 100.

Reference:

1. Kato Y, Kobayashi K, Oda S, Colbourn JK, Tatarazako N, Watanabe H, Iguchi T. Molecular cloning and sexually dimorphic expression of DM-domain genes in *Daphnia magna*. Genomics. 2008;91:94–101
