# Supplementary material for: Sequence Conservation and Sexually Dimorphic Expression of the Ftz-F1 Gene in the Crustacean Daphnia magna
Source: PLoS One. 2016 May 3;11(5):e0154636. doi: 10.1371/journal.pone.0154636 (PMC4854414; doi:10.1371/journal.pone.0154636)
Supplement: S3 File — (DOCX) [file pone.0154636.s003.docx]

**S3 - Supplementary Information**

**The candidates of Ftz-F1-binding site in the *Dsx1* promoter of *D. magna* and *D. pulex.***

To determine the Ftz-F1 binding sites on the promoter region of *DapmaDsx1* gene sequences, we performed computational identification of binding sites using the consensus sequence of Ftz-F1 obtained from [1]. The matrix consensus is shown in Fig 1. We identified nucleotide sequences using RSA-tools Patser program (<http://rsat.ulb.ac.be/patser_form.cgi>) as shown in Table 1. To check whether the binding site sequences are conserved or not in *Daphnia* species, we performed the same computational identification on *Dsx* genes of *Daphnia pulex.* Then, the location of the hypothetical binding sites was mapped in the annotated *Daphnia magna* and *Daphnia pulex Dsx1* gene sequences (Fig 2).

**Figure 1: The matrix of consensus sequence of Ftz-F1 binding site [1].**

**Table 1: The identification of Ftz-F1 binding sites on *Daphnia magna Dsx* gene.**

| **Map** | **Strand** | **Start** | **End** | **Sequence** | **Score** | **ln(P)** |
| --- | --- | --- | --- | --- | --- | --- |
| ftz-f1.m1 | R | -29483 | -29473 | tagtCCAAGGTTGCCacgt | 9.69 | -11.83 |
| ftz-f1.m2 | D | -26569 | -26559 | gcaaCCAAGGCCATCgatt | 9.49 | -11.63 |
| ftz-f1.m3 | R | -23668 | -23658 | tttaCGAAGGCCAACgaat | 8.30 | -10.34 |
| ftz-f1.m4 | R | -13535 | -13525 | aatgGCAAGGACACCaaac | 11.04 | -13.47 |
| ftz-f1.m5 | D | -9340 | -9330 | tttgTCAAGGTCGTCtccc | 8.43 | -10.46 |
| ftz-f1.m6 | D | -4421 | -4411 | acctCCAAGTCCACCatct | 7.78 | -9.87 |
| ftz-f1.m7 | D | -2328 | -2318 | gcatCGAAGGACGAAagac | 7.89 | -9.9 |

D = Direct strand

R= Reverse strand

The lower threshold estimation was 7.7 on the sequence of 31899 bp.

**Table 2: The identification of Ftz-F1 binding sites on *Daphnia pulex Dsx* gene.**

| **map** | **Strand** | **Start** | **End** | **Sequence** | **Score** | **ln(P)** |
| --- | --- | --- | --- | --- | --- | --- |
| ftz-f1.p1 | R | -32740 | -32730 | tagtCCAAGGTTGCCattg | 9.69 | -11.83 |
| ftz-f1.p2 | D | -30058 | -30048 | gcaaCCAAGGCCATCgatt | 9.49 | -11.63 |
| ftz-f1.p3 | D | -28353 | -28343 | gttaCCACGGACACCacca | 9.25 | -11.33 |
| ftz-f1.p4 | D | -27012 | -27002 | ctgaCGAAGGTCCCCcctt | 7.98 | -10.07 |
| ftz-f1.p5 | R | -26801 | -26791 | tttgCGAAGGCCAACcgtc | 8.30 | -10.34 |
| ftz-f1.p6 | D | -24263 | -24253 | gaagCCAAGGATGCTttga | 8.95 | -10.98 |
| ftz-f1.p7 | D | -19508 | -19498 | accaCCACGGCCACCctcc | 8.17 | -10.28 |
| ftz-f1.p8 | R | -2412 | -2402 | gttgCCAAGGACTCTagga | 8.42 | -10.44 |

D = Direct strand

R= Reverse strand

The lower threshold estimation was 7.7 on the sequences of 34798 bp.

**Fig 2: The map of Ftz-F1 binding sites location on *Dsx* gene of *D. magna* and *D. pulex*.**

By comparing both annotations from *D. magna* and *D. pulex*, we found three conserved binding site sequences (m1 with p1, m2 with p2, and m3 with p5) that contained the same “AAGG” core sequence [1]. This result provides us three candidates of *DapmaFtz-F1* binding sites on the promoter region of *Dsx1* gene.

Reference:

1. Bowler T, Kosman D, Licht JD, Pick L. Computational identification of Ftz/Ftz-F1 downstream target genes. Dev Biol. 2006; 299(1): 78-90.
