# Supplementary material for: Sequence Conservation and Sexually Dimorphic Expression of the Ftz-F1 Gene in the Crustacean Daphnia magna
Source: PLoS One. 2016 May 3;11(5):e0154636. doi: 10.1371/journal.pone.0154636 (PMC4854414; doi:10.1371/journal.pone.0154636)
Supplement: S1 Table — (DOCX) [file pone.0154636.s005.docx]

**S1 Table: The number of embryos and amount of isolated total RNA from each males and females sample.**

| **Time** | **Group** | **Male** | | **Female** | |
| --- | --- | --- | --- | --- | --- |
|  |  | **No. of embryos** | **RNA amounts (ug)** | **No. of embryos** | **RNA amounts (ug)** |
| 0 h | 1 | 128 | 4.26 | 184 | 4.63 |
|  | 2 | 128 | 3.82 | 150 | 4.18 |
|  | 3 | 128 | 4.03 | 150 | 3.70 |
| 6 h | 1 | 128 | 3.61 | 162 | 4.80 |
|  | 2 | 127 | 4.06 | 162 | 5.06 |
|  | 3 | 128 | 3.96 | 162 | 3.96 |
| 12 h | 1 | 106 | 3.09 | 208 | 6.29 |
|  | 2 | 107 | 2.95 | 95 | 2.41 |
|  | 3 | 107 | 2.51 | 95 | 2.14 |
| 18 h | 1 | 143 | 8.20 | 188 | 6.75 |
|  | 2 | 99 | 4.93 | 108 | 3.75 |
|  | 3 | 98 | 5.17 | 108 | 3.69 |
| 24 h | 1 | 102 | 7.78 | 153 | 8.14 |
|  | 2 | 102 | 6.21 | 69 | 3.77 |
|  | 3 | 111 | 6.61 | 69 | 3.75 |
| 30 h | 1 | 64 | 4.10 | 148 | 11.19 |
|  | 2 | 79 | 3.89 | 48 | 3.20 |
|  | 3 | 78 | 4.68 | 49 | 3.03 |
| 48 h | 1 | 54 | 5.94 | 50 | 4.63 |
|  | 2 | 53 | 5.46 | 57 | 4.45 |
|  | 3 | 55 | 6.47 | 58 | 5.22 |
| 72 h | 1 | 45 | 8.64 | 48 | 10.13 |
|  | 2 | 43 | 7.14 | 52 | 11.02 |
|  | 3 | 48 | 9.00 | 51 | 11.93 |
